# Automated serum chloride analysis using the Apple computer

**DOI:** 10.1155/S1463924688000057

**Published:** 1988

**Authors:** Paul J. Taylor, Rosalie A. Bouska

**Affiliations:** 1 Chemistry Department University of Wisconsin-La Crosse La Crosse Wisconsin 54601 USA; 2 RTP Co. 530 E. Front Street P.O. Box 439 Winona Minnesota 55987 USA

## Abstract

Chloride analysis employing a coulometric technique is a wellestablished method. However, the equipment needed is specialized and somewhat expensive. The purpose of this paper is to report the development of the hardware and software to perform this analysis using an Apple computer to control the coulometric titration, as well as to automate it and to print out the results.

The Apple computer is used to control the flow of current in a circuit, which includes silver and platinum electrodes where the following reactions take place:$Ag\to Ag^{+}+le^{-}\left(\text{at silver anode}\right)$ $2H_{2}O+2e^{-}\to 2OH^{-}+H_{2}\left(\text{at platinum cathode}\right)$

The generated silver ions then react with the chloride ion in the sample to form AgCl.$Ag^{+}+Cl^{-}\to AgCl_{\left(s\right)}$

When all of the chloride ion has been titrated, the concentration of silver ions in solution increases rapidly, which causes an increase in the current between two silver microelectrodes. This current is converted to a voltage and amplified by a simple circuit. This voltage is read by the analogue-to-digital converter. The computer stops the titration and calculates the chloride ion content of the sample. Thus, the computer controls the apparatus, records the data, and reacts to the data to terminate the analyses and prints out the results and messages to the analyst.

Analysis of standards and reference sera indicate the method is rapid, accurate and precise. Application of this apparatus as a teaching aidfor electronics to chemistry and medical students is also described.


Journal of Automatic Chemistry, Vol. 10, No. 1 (January–March 1988), pp. 10–14

### Automated serum chloride analysis using the Apple computer*

**Paul J. Taylor and Rosalie A. Bouska**[1]

*Chemistry Department, University of Wisconsin-La Crosse, La Crosse, Wisconsin 54601, USA*

*Chloride analysis employing a coulometric technique is a well-established method. However, the equipment needed is specialized and somewhat expensive. The purpose of this paper is to report the development of the hardware and software to perform this analysis using an Apple computer to control the coulometric titration, as well as to automate it and to print out the results.*

*The Apple computer is used to control the flow of current in a circuit, which includes silver and platinum electrodes where the following reactions take place:*

$$Ag \rightarrow Ag^+ + le^- \text{ (at silver anode)}$$

$$2H_2O + 2e^- \rightarrow 2OH^- + H_2 \text{ (at platinum cathode)}$$

*The generated silver ions then react with the chloride ion in the sample to form AgCl.*

$$Ag^+ + Cl^- \rightarrow AgCl_{(s)}$$

*When all of the chloride ion has been titrated, the concentration of silver ions in solution increases rapidly, which causes an increase in the current between two silver microelectrodes. This current is converted to a voltage and amplified by a simple circuit. This voltage is read by the analogue-to-digital converter. The computer stops the titration and calculates the chloride ion content of the sample. Thus, the computer controls the apparatus, records the data, and reacts to the data to terminate the analyses and prints out the results and messages to the analyst.*

*Analysis of standards and reference sera indicate the method is rapid, accurate and precise. Application of this apparatus as a teaching aid for electronics to chemistry and medical students is also described.*

### Introduction

The analysis of chloride in body fluids, especially serum, is routinely done. Low serum chloride values are seen in salt-losing, nephritis, during Addisonian crisis, diabetic acidosis and renal failure. High serum chloride values are possible during dehydration, hyperchloremic renal acidosis and in conditions causing decreased renal blood flow, such as congestive heart failure [1 and 2].

A number of methods have been developed for the determination of chloride in serum and other body fluids [1–10]. These include:

(1) Titration with $Hg^{2+}$ using diphenylcarbazone as the end-point indicator.
(2) An autoanalyzer method employing excess $Hg(SCN)_2$. The thiocyanate released when the mercury reacts with chloride ion reacts with iron(III) to form a coloured complex, $Fe(SCN)_3$.
(3) A coulometric-amperometric titration where silver ion, $Ag^+$, is generated at a silver electrode and allowed to react with chloride ion.
(4) An automated method based on the formation of a coloured complex between Ferric perchlorate and chloride ion.
(5) Methods employing ion selective electrodes.
(6) Ion chromatography.

Horvai *et al.* [4] used an electrochemical detector with ion chromatography to analyse for chloride ion.

Several patents have been applied for or awarded for devices which analyse for chloride ion. Lower concentrations of chloride ion should be determined coulometrically by using a standard addition technique [5]. Natelson [6] developed a multilayer test strip to assay for several substances simultaneously. This included chloride ion using an ion-specific electrode. Blanke [7] used a device employing a Ag coulometric electrode and two smaller Ag detector electrodes to determine chloride ion in serum. A device has been developed [8] which compares the chloride concentration in the sample to the standards and adjusts the sample size fed into the measurement system when the chloride content in the sample is outside the range provided by the standards.

Pre [9] *et al.* developed a method for the simultaneous electrochemical measurement of chloride ion, total carbon dioxide, and sodium and potassium ion in blood plasma using a two electrode apparatus.

Using an indicator Ag electrode and a $Hg/Hg_2SO_4$ reference electrode Sliwinska [10] developed a method to coulometrically titrate chloride ion in serum.

Bender [2] has developed a manual method to coulometrically titrate chloride ion. At a platinum cathode water is reduced to $OH^-$ which is removed by a $HNO_3/HAc$ buffer. At a silver anode $Ag^+$ ions are generated which precipitates the chloride as AgCl. The end-point is detected amperometrically with two indicator electrodes. A pair of micro-silver electrodes with 0·1 to 0·2 V applied across them is placed in the solution. Since the current between these detector electrodes is proportional to the silver ion content, a rapid rise in current

* This paper presented in part at the 1986 Pittsburgh Conference on Analytical Chemistry and Applied Spectroscopy, Atlantic City, New Jersey, 10–14 March, 1986.

[1] Current address RTP Co., 530 E. Front Street, P.O. Box 439, Winona, Minnesota 55987, USA.

signals the end-point. The method described in this paper is an automated computer controlled procedure based on this manual method.

Our method performs coulometric titrations of chloride ion in serum or plasma in a manner similar to commercially available devices. No special equipment is needed, however. Both the Apple computer and interface board are general purpose and can be used for other purposes when not being used for the analysis of chloride ion. Also, the computer can be used to print out results and store analysis data on the disk, features not possible on some commercial units. The electrochemical cell and interface are inexpensive.

### Experimental

*Reagents and Standards*

Sodium chloride, ACS, was purchased from EM Science; nitric acid was purchased from Fisher; acetic acid thymol and thymol blue were purchased from J. T. Baker; gelatin, unflavoured, was purchased from Knox Gelatin Inc.; the silver electrode (No. 476065) was purchased from Corning and the platinum electrode (No. 39273) was purchased from Beckman. Freeze-dried Bovine and Human serum reference samples were purchased from General Diagnostics (Division of Warner-Lambert Co., Morris Plains, New Jersey 07950, USA).

*Preparation—reagents were prepared as per Bender* [2]

Supporting electrolyte, 0.1N nitric acid and 1·8M acetic acid.

*Gelatin reagent:* Weigh out 0.60 grams of Knox unflavoured gelatin, 10 mg. Thymol blue and 10 mg Thymol. Dissolve this mixture in 100 ml of boiling water. Divide this into ten 10 ml vials and refrigerate. The reagent is stable for six months when refrigerated.

Chloride standard, 100 meq/litre.

Freeze-dried serum samples were reconstituted with diluent as per the manufacturer's directions. When not in use, they were refrigerated. They were discarded after 24 h.

*Electrodes*

The platinum cathode and silver anodes were used as received. The silver detector electrodes were made by soldering leads to 4 cm pieces of silver wire and sealing them into a piece of 10 cm long by 5 mm I.D. glass tubing with epoxy glue [2].

*Interface*

Circuit components used to build the interface between the electrodes and Apple computer are: Light Activated Silicone Controlled Relay (LASCR), Potter & Bramfield, operational amplifiers; 741 Radio Shack 276-007, VN10KM, Radio Shack 276-2070, DPDT DIP Relay, Radio Shack 275-215, Hex inverter, National semiconductor.

The Addalab® board was purchased from Interactive Microware, State College, Pennsylvania, USA.

*Procedure*

Place 10 ml of supporting electrolyte and 500 μl of gelatin reagent in the cell. Set up the electrodes and stirrer. Run the program CL TITER. Add 100 μl of a suitable standard or serum sample when directed by the software.

CL TITER is a program written in BASIC, which runs the experiment and prints out the results. A copy of this program can be obtained by sending a blank floppy diskette to the authors.

The supporting electrolyte is used to maintain an acidic solution and prevent the precipitation of silver hydroxide from the hydroxide ion generated at the platinum electrode. Gelatin or polyvinyl chloride is added to prevent reduction of silver chloride at the indicating electrodes and to promote uniform deposition of excess silver ions on the indicator cathode [3]. Thymol blue is used to make sure the vials of gelatin are acidic and thymol is a preservative [2].

### Results

The interface between the electrochemical cell and the Apple II computer is shown in figure 1. Bit 1 of the digital output of the Addalab board is used to control the LASCR which turns the magnetic stirrer on and off. The 741 op-amp is wired as a follower to provide the current to activate the LASCR. Without this op-amp, bit 1 will drop below 5 V when high and, thus, not activate the relay.

When bit 2 goes high, the output of the Hex inverters goes low (ground), which then activates the DPDT relay connecting the constant current generator to the cell. The current needed to operate the relay is drawn from the inverters, rather than directly from the digital output port.

A voltage drop of about 0·2 V is maintained across two micro-silver electrodes. The current between these two electrodes is directly proportional to the silver ion concentration in solution. The analogue-to-digital converter (ADC) can only monitor voltage signals. Thus, a current to voltage conversion is necessary. This is accomplished by causing the detector current to flow through a 10K resistor. The voltage drop produced is then amplified by a factor of 10 and read by the ADC.

In the manual method, to coulometrically titrate chloride ion [2], a constant current generator is connected between the silver and platinum electrodes. The current between the detector electrodes is monitored, and when it increases 10 μA above the value established at the start of the titration, the constant current generator is disconnected from the working electrodes. A stop-watch is used to measure the time of current flow. Each titration is, thus, titrated beyond the equivalence point. The procedure is repeated using a reagent blank. The time needed to titrate the blank is subtracted from time needed to titrate the sample to correct for the over-titration. Each

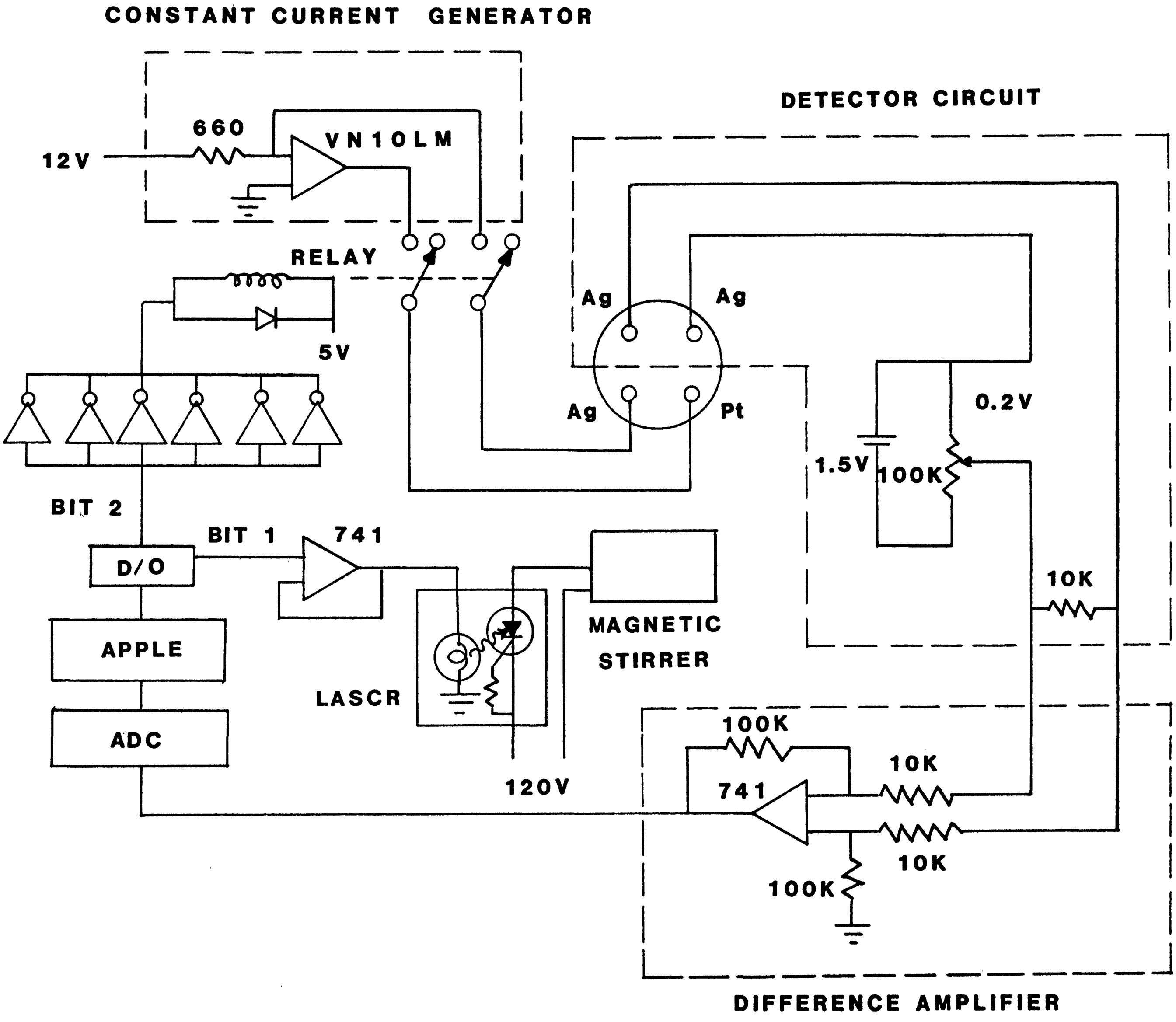

*Figure 1. Schematic diagram of titrator interface.*

run in the manual method required cleaning the cell and electrodes and adding fresh reagents. Fresh reagents are necessary because each sample is titrated beyond the end point. Thus, unreacted $Ag^+$ is present, which would produce low results for a subsequent analysis if fresh reagents were not used and the cell not cleaned.

The method initially mimicked the manual method. A typical graph of current flow in the detector circuit versus data point number is shown in figure 2. Data points were taken every 0·60 s. This figure indicates that the true equivalence point is at the intersection of the base current and the rapidly rising current beyond the equivalence point. In an attempt to stop the titration at the equivalence point, and not to over-titrate the sample, the constant current generator was disconnected when the detector current was 0·5 μA higher than the minimum value. However, depending on the sample, this could cause a small over-titration or under-titration as is shown in figure 3. Point C is the equivalent Point. Point A is

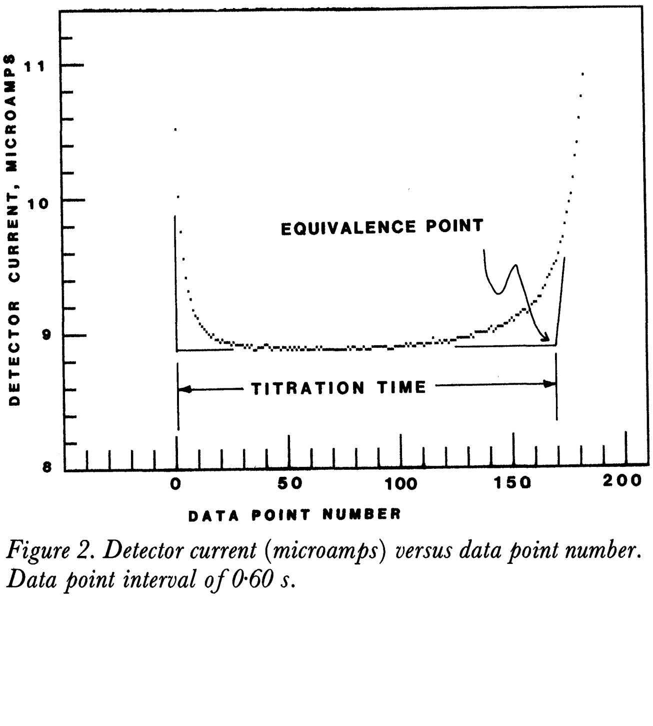

*Figure 2. Detector current (microamps) versus data point number. Data point interval of 0·60 s.*

where this titration would have been stopped using this method, and would have under-titrated this particular sample by a small, but measureable amount. This would have produced a low result for this sample.

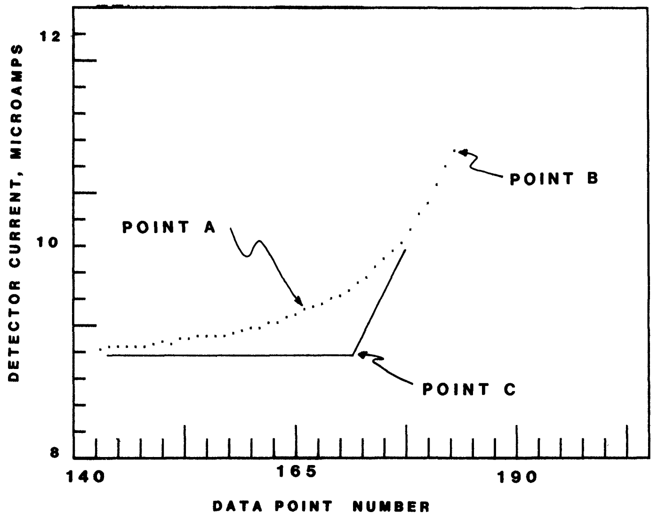

*Figure 3. Detector current (microamps) versus data point number near equivalence point. Data point interval of 0·60 s.*

An improved procedure called for titration to the point where the detector current was on the linearly rising portion of the titration curve, 2·0 μA greater than the minimum value was used to indicate the end-point. Because this point, point B in figure 3, is on the linearly rising portion of the curve it is more reproducible. Each sample is over-titrated by the same amount which is represented by the extra time (and current) needed to get from Point C to Point B in figure 3. The excess silver ion generated is then available to react with a subsequent sample which is also over-titrated to Point B. This permits one to titrate numerous samples without cleaning the cell and using fresh supporting electrolyte and other reagents for each sample.

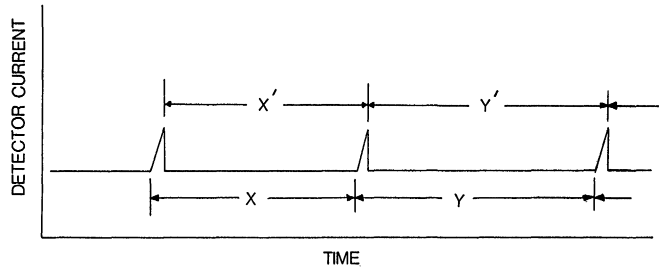

*Figure 4. Detector current versus time for successive samples.*

Figure 4 depicts the titration of several successive samples. Because the extent of over-titration of each sample is the same, the time between end-points ($x'$ or $y'$) is the same as the time between equivalence points ($x$ or $y$). Dozens of successive samples have been titrated in this manner with no loss in accuracy or precision. The first analysis, however, must be discarded, because of over-titration and because there is no way of correcting for the over-titration. All of the results reported here were done using this method.

The computer program is written in BASIC and well documented. Operating instructions are provided to inform the operator when to add reagents, or sample. During the titration, the time versus detector current is displayed on the screen in tabular format. When the titration is complete this table may be printed out, or simply the results may be reported as time for titration and milliequivalents of chloride per litre.

### Conclusions

The results of the analysis of standards are shown in table 1 and graphed in figure 5. The statistical data indicates that the calibration curve is linear and very well behaved. The slope (1·0039) and intercept (0·192) are exceptionally close to the expected values of 1 and 0, respectively. Analysis of freeze-dried human and bovine reference serum (General Diagnostics) are shown in table 2. The data indicate that the experimentally determined values are in good agreement with the known values. The discrepancies between the experimental and known values were positive in both cases; the discrepancies for the human serum were smaller than those of the bovine serum. The results of the analysis of a serum sample by the standard addition techniques are shown in table 3 and

*Table 1. Analysis of standards, MEQ/l.*

| Standard | Result | *N* | s |
|---|---|---|---|
| 50·00 | 50·52 | 9 | 1·2 |
| 80·00 | 81·15 | 9 | 1·6 |
| 100·00 | 99·42 | 8 | 0·99 |
| 150·00 | 151·17 | 10 | 1·6 |

N = Number of samples.

s = Standard deviation.

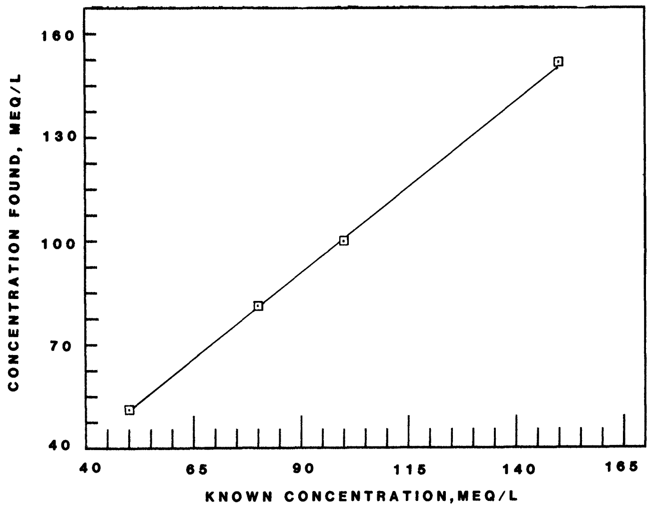

*Figure 5. Concentration found, MEQ/l versus known concentration, MEQ/l for NaCl standards. Slope = 1·004; intercept = 0·192; coefficient of correlation = 0·9998; coefficient of determination = 0·9996.*

*Table 2. Analysis of serum samples.*

| | |
|---|---|
| Bovine serum | |
| Known value | 100·0 MEQ/l |
| Experimental value[a] | 102·6 MEQ/l |
| Human serum | |
| Known value | 122·0 MEQ/l |
| Experimental value[b] | 122·7 MEQ/l |

(a) 26 samples; standard deviation 3·2.
(b) 61 samples; standard deviation 4·0.

*Table 3. Results of serum analysis, MEQ/l.*

| Sample | Mean | $N$ | s |
|---|---|---|---|
| X | 112·8 | 10 | 1·9 |
| X + 50 | 162·3 | 5 | 4·4 |
| X + 80 | 189·3 | 5 | 4·3 |
| X + 100 | 207·9 | 4 | 0·9 |

$N$ = Number of samples.

s = Standard deviation.

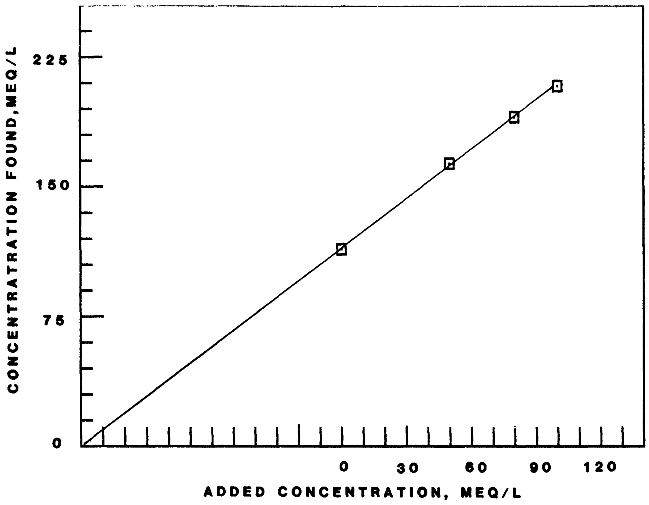

*Figure 6. Concentration found, MEQ/l versus added concentration, MEQ/l for freeze dried serum sample. Slope = 0·950; intercept = 113·45; coefficient of correlation = 0·9997.*

graphed in figure 6. The slope (0·95009) of this plot indicates that at high concentrations of chloride a small negative error exists. This may be a result of incomplete reaction between the chloride and generated silver ion in these solutions. These samples are significantly more concentrated in chloride than normal serum samples. Normal serum samples would produce results with smaller errors, as indicated in figure 5 where the slope is nearer to one.

The procedure is rapid (1 to 2 min per sample) convenient, accurate and precise. This method can, therefore, be used to replace existing manual methods or as an alternative to expensive and specialized coulometric titrators. It is used in the authors' instrumental analysis courses for chemistry majors and medical technology majors to teach digital electronics and computer automation.

The student gains experience with digital and analogue electronics (see figure 1). Methods to drive analogue devices from a digital computer are introduced. Several applications of operational amplifiers, a basic building block in modern electronics, are employed. The students perform this experiment after first being introduced to BASIC programming. He/she is then in a position to understand and explain how the program operates and performs the automated coulometric titration. Using this hand-built apparatus enables the student to understand how the commercially available equipment functions. The 'black-box' mystique of these devices is therefore minimized.

### References

1. Tietz, N. W., in *Fundamentals of Clinical Chemistry,* Tietz, N.W. (Ed.) (Saunders, Philadelphia, 1982), p. 880.
2. Bender, G. T., *Chemical Instrumentation: A Laboratory Manual Based on Clinical Chemistry* (Saunders, Philadelphia, 1972), p. 223.
3. Miller, W. G., in *Clinical Chemistry,* Kaplan, L. A. and Pease, A. J. (Eds) (C. V. Mosby, 1984), p. 1059.
4. Horvai, G., Fekete, J., Niegreisz, Z., Toth, K., and Pungor, E., *Journal of Chromatography,* **385** (1987), 25–32.
5. Japanese Patent 171853, 1984; *Chemical Abstracts,* **102**, 4 (1985), 39150y.
6. Natelson, S., US Patent 4264560, 1981; *Chemical Abstracts,* **95**, 3 (1981), 20879h.
7. Blanke, G. C., US Patent 4230554, 1980; *Chemical Abstracts.,* **94**, 11 (1981), 79870f.
8. Japanese Patent 91356, 1984; *Chemical Abstracts,* **101**, 18 (1984), 162916j.
9. Pre, J. and Huet, B., *Feuill. Biol.,* **21**, 115 (1980), 49–53.
10. Sliwinska, J., *Diagnostic Laboratory,* **13**, 5 (1977), 292–4.

